# Confederate Medical and Surgical Journal

**Published:** 1864-05

**Authors:** 


Confederate Medical and Surgical Journal.—The *London Lancet* states that it has received the first number of what it believes is the first medical periodical published in the South, having the above title. "It is meant not only as the organ of the Southern medical profession, but as a means of imparting information to those who have been debarred from any intercourse with the scientific world." The contents are:—A paper on traumatic tetanus, by Prof. J. Jones; another on resections of the hip; and a third on the external application of the oil of turpentine as a substitute for quinine in intermittent fever; the remainder is of local interest. The place of publication is not given. We cordially unite with the *Lancet* in expressions of sympathy for "our medical brethren in the South," and trust the time is not distant when they will cease to walk in the ways of transgression.—*American Medical Times.*

